# Scaling behavior of public procurement activity

**DOI:** 10.1371/journal.pone.0260806

**Published:** 2021-12-08

**Authors:** António Curado, Bruno Damásio, Sara Encarnação, Cristian Candia, Flávio L. Pinheiro

**Affiliations:** 1 NOVA Information Management School (NOVA IMS), Universidade Nova de Lisboa, Lisboa, Portugal; 2 Instituto Superior de Economia e Gestão (ISEG) - Universidade de Lisboa, Lisboa, Portugal; 3 Interdisciplinary Centre of Social Sciences (CICS.NOVA), Faculty of Social Sciences and Humanities (FCSH/NOVA), Lisboa, Portugal; 4 ATP-Group, Porto Salvo, Portugal; 5 Data Science Institute, Facultad de Ingeniería, Universidad del Desarrollo, Las Condes, Chile; 6 Kellogg School of Management, Northwestern University, Evanston, IL, United States of America; 7 Northwestern Institute on Complex Systems (NICO), Northwestern University, Evanston, IL, United States of America; 8 Centro de Investigación en Complejidad Social (CICS), Facultad de Gobierno, Universidad del Desarrollo, Las Condes, Chile; Northwest University, UNITED STATES

## Abstract

Public procurement refers to the purchase by public sector entities—such as government departments or local authorities—of Services, Goods, or Works. It accounts for a significant share of OECD countries’ expenditures. However, while governments are expected to execute them as efficiently as possible, there is a lack of methodologies for an adequate comparison of procurement activity between institutions at different scales, which represents a challenge for policymakers and academics. Here, we propose using methods borrowed from urban scaling laws literature to study public procurement activity among 278 Portuguese municipalities between 2011 and 2018. We find that public procurement expenditure scales sublinearly with population size, indicating an economy of scale for public spending as cities increase their population size. Moreover, when looking at the municipal Scale-Adjusted Indicators (the deviations from the scaling law) by contract categories—Works, Goods, and Services—we are able to identify a richer local characterisation of municipalities based on the similarity of procurement activity. These results make up a framework for quantitatively studying local public expenditure by enabling policymakers a more appropriate foundation for comparative analysis.

## Introduction

Public procurement contracts—defined by the OECD as the purchase by governments and state-owned enterprises of goods and services [1]—are an essential public sector instrument allowing policymakers to push-forward inclusive socio-economic standards [2], promote innovation and economic growth [3–5] policies effectively. Among OECD countries, public procurement weighs, on average, 29% of all governmental expenditures [2] (14% among EU countries [6]) and 12% of global OECD countries GDP. Moreover, given the relevance of the economic activity of public procurement mechanisms, the European Commission has established a common framework for public procurement aimed at ensuring *equal treatment and transparency, reduce fraud and corruption and remove legal administrative barriers to participation in cross-border tenders* [7]. Furthermore, data on public procurement contracts should also constitute the basis for analytical frameworks that effectively evaluate the effectiveness and impact of public sector activities at different scales and dimensions comparatively. However, few studies have explored adequate methodologies that account for non-linearities in spending dynamics or examined the inference potential in public procurement data [8–10].

Here, we propose using methods borrowed from urban scaling laws literature, which are rooted in statistical physics and complexity sciences, to characterize municipal public procurement activity. Urban scaling laws [11–14] have been widely used across different disciplines to describe the relationship between socio-economic indicators in relation to the size of population agglomerates. The resulting literature prompted a revision of current urban planning frameworks and comparative indicators [15] while leading researchers to look for universal laws in cities and urban growth. In that sense, applying these methods to study public procurement activity can, in our view, lead to a similar revision on the current frameworks to evaluate regional public procurement policies and quantify their activity.

Urban scaling laws model the relationship between an indicator, *Y*, and the size (e.g., population, area)—*X*—of a set of sectional units (*e.g*., cities or urban areas) as a power-law, that is

$$\begin{array}{c} Y∼\alpha X^{\beta } \end{array}$$ (1)

where *β* is the scaling factor and *α* represents the natural baseline activity of a region [16, 17]. In the context of urban scaling laws, several indicators—such as water consumption, housing, or jobs [18]—have been shown to follow a linear relationship (*β* = 1.0). However, the more interesting cases are those in which *Y* exhibits a superlinear (*β* > 1) or sublinear (*β* < 1) relationship with *X*. Such cases identify particular indicators that either scale above (superlinear) or below (sublinear) linear growth with increasing population size. superlinear behavior is often observed in the regional economic output [19–25], energy consumption and pollution [26–28], employment [29, 30] criminality [30–34], number of patents [25, 29, 33, 35], wages [33], employment in R&D [33] and urbanized areas [29]. Examples of sublinear relationships are found in the total length of road networks [21, 33] and power grids [36]. In other cases, like supply networks, *Y* exhibits sub or superlinear behavior depending on the industry [36, 37], and voter turnout [38].

It should be noted that the present analysis at the municipality level is not directly comparable with past works in urban scaling laws literature [39], which have focused on studying cities from a functional perspective [12, 18, 40–42]. Instead, and due to the local scale nature of procurement activities, we focus on the administrative boundaries of city governance in Portugal, *i.e*., namely municipalities [43, 44]. Moreover, while past works explore how the population size of a city impacts its function in different dimensions (e.g., human, cultural, and innovation outputs but also industry prevalence and infrastructure costs), our study explores an administrative dimension of cities through public expenditure.

Municipalities constitute an interesting intersection between a regional unit of governance and a population agglomerate [43, 44]. They are also units at which policy related questions to procurement activity, in terms of sustainability goals and execution efficiency, are particularly relevant [45–48]. In Portugal, Municipalities are a Local Administrative Unit [49], which form the building blocks of the NUTS (Nomenclature of Territorial Units for Statistics) European regional units representing the second-largest administrative division whose governance body is elected by universal suffrage. They are also the administrative division with the most stable regional boundaries and upon which city governance responsibility falls. Thus being, a suitable candidate to study the scaling behavior of procurement activity.

It is also worth emphasizing that the volume of procurement activity, at the municipality level, is closely linked with the size of the local economy. That is, municipalities with larger budgets, for instance, will spend proportionally more on public procurement contracts. Such a relationship, which we show in the SI, exhibits a high correlation coefficient (around 0.86) and thus opens the question of whether procurement data is but a proxy for the size of the economy. However, the study of procurement data at the municipal level, in and of itself, has inherent value for several reasons: 1) it provides a more fine-grained categorization of the expenditure dynamics of municipalities, as contracts detail the nature of the service being contracted, both from the supply and demand side, offering detailed information about private companies also, unlike budgetary data; 2) due to international agreements aimed at increasing market efficiency and competitiveness (as is in the case of EU), they are standardized across countries and thus provide a unique opportunity to compare regional activities—this circumstance is not possible with budgetary data [7]; 3) public procurement contracts also have higher transparency than budgetary data, as the latter is often opaque with accounting procedures that change according to national laws and are thus different across regions [50]. Furthermore, budgetary data depends also on institutional constraints that vary over time and oscillate with the political cycles [51]); 4) it is a dimension in which there is a high risk of anomalies, fraud, and corruption, and as such, there is a need for the development of accurate models of what constitutes an expected level of procurement activity and dynamics to identify suspicious activities [52–54]; and 5) unlike budgets, which are not always consolidated, public procurement data also provide us information about the expenditure dynamics of public companies and inter-municipal companies.

We start with a description of the data sources and data pre-processing steps. Then, we characterize municipality procurement activity from an urban scaling law perspective, showing that procurement activity scales sublinearly with population size. We then use Scale-Adjusted Indicators (measured as the deviations/residuals from the scaling laws, a measured also known as SAMI in USL literature [11]) to quantify the differences in procurement activity between different regions (groups of municipalities), but also to obtain a new regional characterization of municipalities through their similarities in revealed procurement activity. We conclude with final remarks and a discussion of future working directions.

## Data

We used data on Portuguese public procurement contracts sourced from the open-access governmental portal BASE (base.gov.pt). BASE is a public repository managed by the *Instituto dos Mercados Públicos, do Imobiliário e da Construção* (IMPIC) and results from the efforts of the Portuguese government to comply with European open data policies established in 2004 [55]. Since 2008 and by decree [56], public administration bodies have published their procurement activity online through BASE [57].

The working data set comprises 930, 513 contracts issued between January 2009 and December 2018. Each contract relates an issuer that acquires services/goods/works from a supplier and contains information on the issue date; the value of the contract (in euros); category of contract; and Fiscal Numbers of both the issuer and supplier. We analyzed contracts issued by the 278 Municipalities that constitute mainland Portugal (municipalities in the Azores and Madeira archipelagos have not been considered as they represent autonomous administrative regions). Since municipalities can also constitute municipal firms (i.e., a municipality can be the single shareholder of another firm), we aggregated all municipalities and respective child firms into a single entity. The aggregation was manually curated with support from the Annual Financial Booklet of Portuguese Municipalities [58–60]. The pre-processing steps included:

1. Removing observations with contract values equal to or smaller than one;
2. Identifying the Fiscal Number of each Municipality to use them as a primary key;
3. Aggregating municipal firms to the parent Municipality;
4. Discarding all non-Municipality related procurement contracts;
5. The value of contracts that involved more than one municipality were split equally among all participating municipalities (the issuers).

**Fig 1:**
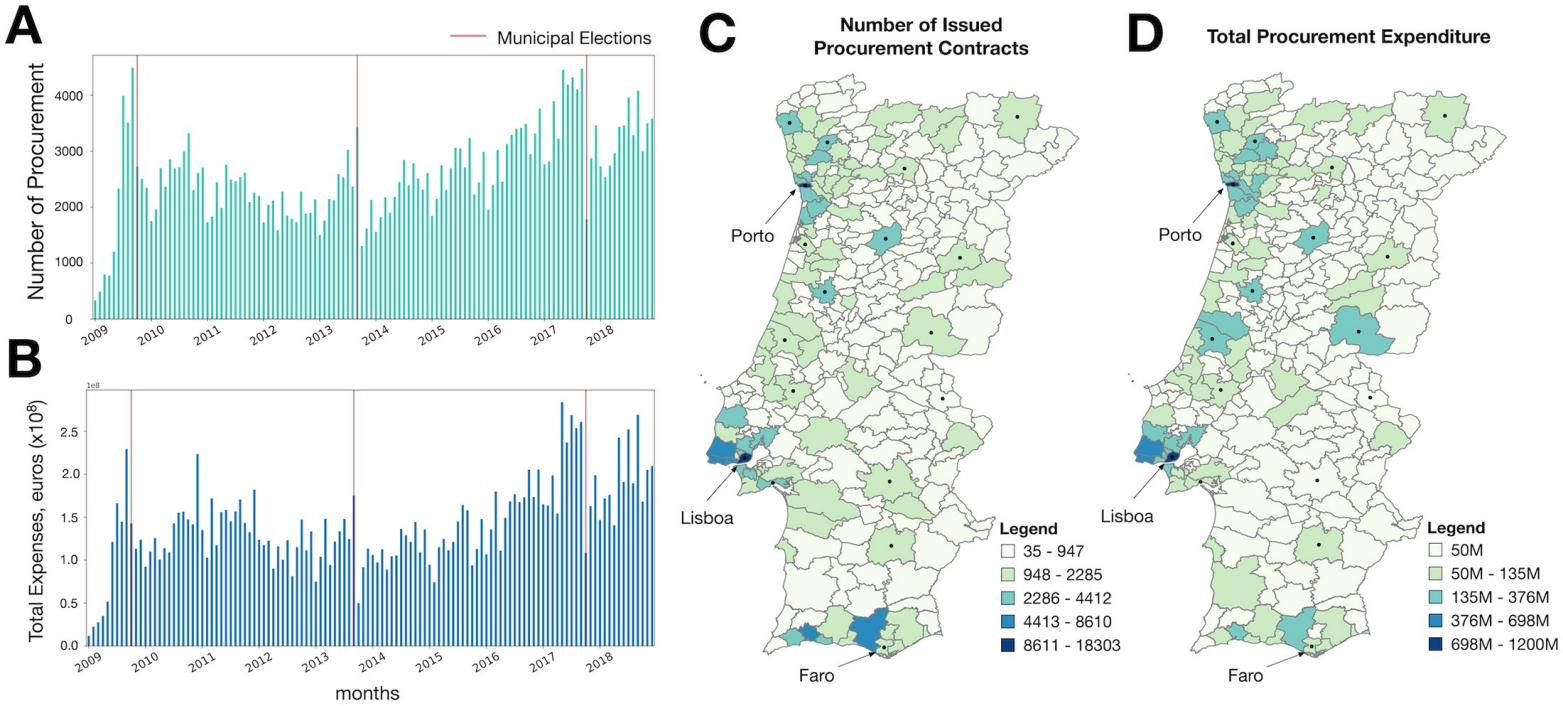
(A) Number of monthly procurement tenders issued between 2009 and 2019 by Portuguese municipalities. (B) Total value in euros derived from procurement, monthly issued by Portuguese municipalities between 2009 and 2019. (A,B) Bars correspond to a month/year, and vertical red lines indicate Municipal elections held nationwide. (C) Spatial distribution of the total number of issued procurement tenders by a municipality. (D) Spatial distribution of the total value spent in procurement contracts per municipality between 2009 and 2019.

The final dataset comprises 310, 819 contracts totaling a value of 16.9 billion Euros. Fig 1A shows the monthly number of contracts issued, while panel b) shows the total value of those contracts. Through visual inspection, it is possible to identify a tendency for municipalities to increase the number of procurement contracts issued in the months leading to elections (red vertical lines in Fig 1A. However, the same does not necessarily translate into an increase in expenditure. Fig 1C and 1D show the spatial distribution of the total number of contracts (Fig 1C) and the total value per municipality (Fig 1D).

Each procurement is associated with the category of contract it represents, which follows the standard classification from the European Commission [7]: Work contracts designate contracts whose execution and/or design include civil engineering works such as roads or sewage plants; Goods contracts identify contracts that are associated with the purchase, lease, or rental of products such as vehicles or computers; Service contracts involve all contracts that have as an object the provision of services such as consultancy, training, or cleaning services. Hence, we computed the total expenditure in procurement contracts per year for each municipality, as well as the total expenditure by procurement contract category. Moreover, since the annual expenditure was rather noisy, we applied a sliding window technique (moving average) of three years. In that sense, the procurement values at year *t* correspond to an average of the values from years *t* − 2, *t* − 1, and *t*. The reported noise can have multiple sources. For instance, a municipality might have issued a procurement contract for the execution of construction in one year that was reflected in the forthcoming annual budgets and thus decreased its construction activity in the following years.

Finally, we enriched the data set with additional indicators by municipality and year. From Pordata [61] we sourced data on Social Integration Income; House Prices; Number of Public Workers; Total Births; Number of Large Corporations; Number of Divorces; Amount of Credit; Number of Medical Doctors; Number of Cultural Events Attendees; Imports and Exports Volume; and Environment Expenses. While from INE [62] we sourced ATM Withdrawals, Municipal Property Tax, Volume of Business in Accommodation, Catering, and Retail; Individual Gross Income; Average Salary of Full-Time Workers. We use these indicators to provide a point of reference to the analysis of public procurement activity, while allowing us to compare with previous urban scaling laws literature.

## Results and discussion

### Scaling laws of municipal procurement expenditure

**Fig 2:**
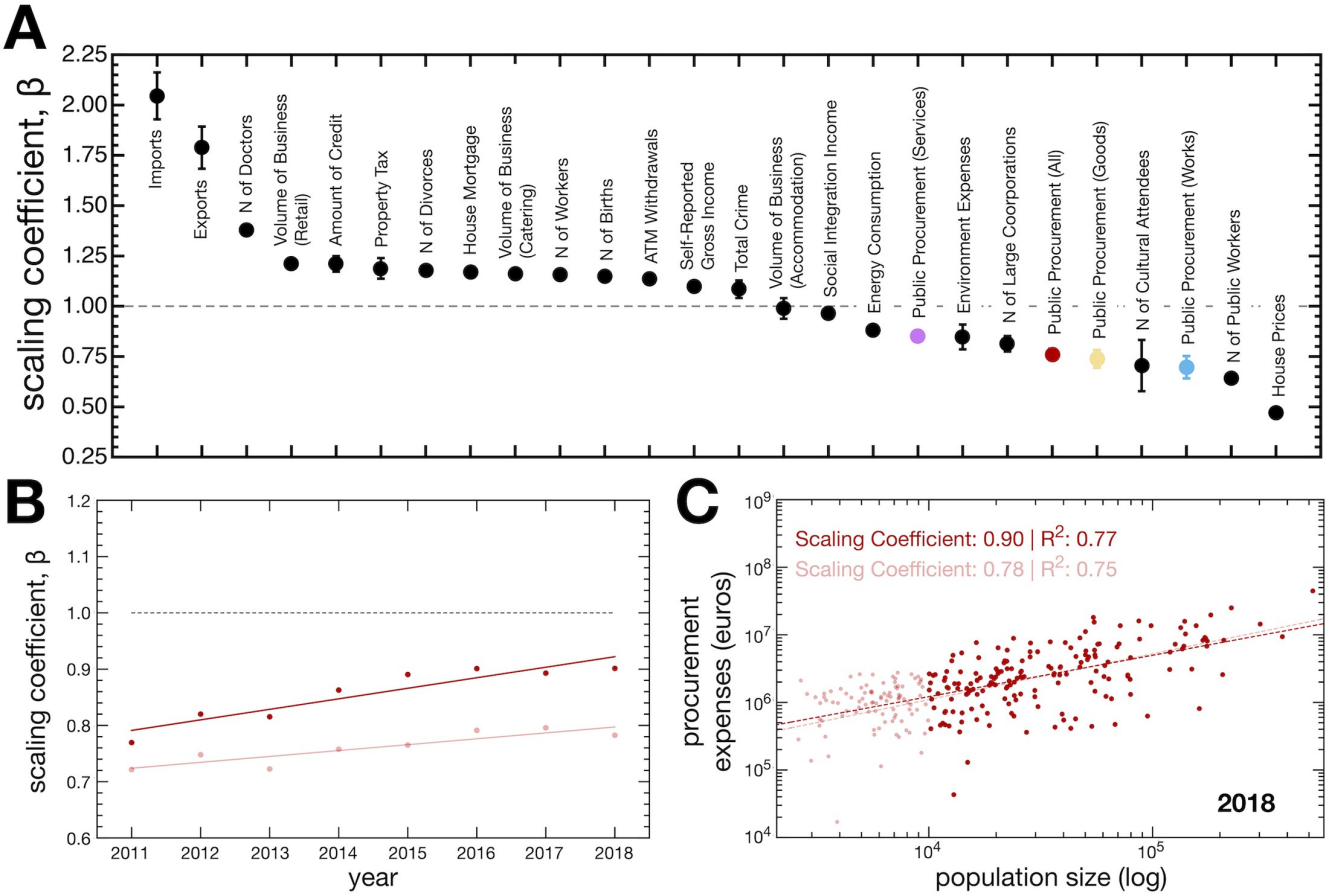
(A) Average scaling coefficients for multiple socio-economic metrics (dark) and all procurement expenses (red). Moreover, we also show the coefficients obtained for the different categories of procurement contracts: works (blue), goods (yellow), and services (purple). Error bars indicate the standard deviations of estimated coefficients for the different years. (B) shows the annual changes in the scaling coefficients for procurement activity. (C) exemplifies the identified relationships between the procurement expenditure (euros) and population size for the year 2018. (B and C) results for municipalities with more than 10^4^ residents are highlighted in dark red, light red indicates the results obtained when considering all municipalities.

We started by comparing the estimated scaling coefficients from municipal procurement activities with those estimated from an extensive set of socio-economic indicators (Fig 2A). The coefficients were estimated independently for each year between 2011 and 2018. Fig 2A shows the average coefficient (Y-Axis) per indicator (X-Axis) with error bars representing the standard deviation.

In general, the obtained scaling coefficients are inline with previous findings in the urban scaling laws literature, thus supporting the choice of analysis at the municipality level. Namely, a superlinear behavior was observed for the volume of imports (*β* = 2.05 ± 0.12) and exports (*β* = 1.79 ± 0.10), number of medical doctors (*β* = 1.38 ± 0.01), the volume of business from retail except for car sales (*β* = 1.21 ± 0.01), amount of credit (*β* = 1.21 ± 0.04), municipal property tax collected (*β* = 1.19 ± 0.05), number of divorces (*β* = 1.18 ± 0.02), total volume of house mortgages (*β* = 1.17 ± 0.02), volume of business from catering (*β* = 1.16 ± 0.03), number of workers (*β* = 1.16 ± 0.01), number of births (*β* = 1.15 ± 0.02), ATM withdrawals (*β* = 1.14 ± 0.01), self-reported gross income (*β* = 1.10 ± 0.01), and reported crime (*β* = 1.08 ± 0.04). Linear scaling was observed for total the volume of business from accommodation (*β* = 0.99 ± 0.05). Sublinear scaling was observed for energy consumption (*β* = 0.88 ± 0.006), social integration income (*β* = 0.97 ± 0.03), environment expenses (*β* = 0.84 ± 0.06), number of large corporations (*β* = 0.81 ± 0.04), number of cultural event attendees (*β* = 0.70 ± 0.13), number of public workers (*β* = 0.70 ± 0.06), and house prices (*β* = 0.47 ± 0.02).

Fig 2B and 2C explore the results obtained from the total procurement expenses per municipality in more detail. Fig 2B shows the annual change in the scaling coefficient, which exhibits an upward temporal trend. Light-colored points indicate scaling coefficients estimated when considering all municipalities, while dark-colored ones only consider municipalities with a population size larger than 10^4^. Fig 2C shows a representative example of the scaling behavior from the year 2018. The threshold was set to filter out low populated municipalities with small procurement activity, and does not reflect any underlying administrative differences between municipalities (we discuss the sensibility of our results to the choice of threshold more in detail below). In that sense, Fig 2B and 2C show the impact of including (lighter color) or not including (darker color) municipalities with a population lower than 10^4^. In all cases, the coefficient shows a sublinear relationship between the total public procurement expenditure and population size.

### Scaling of procurement activity by contract category

**Fig 3:**
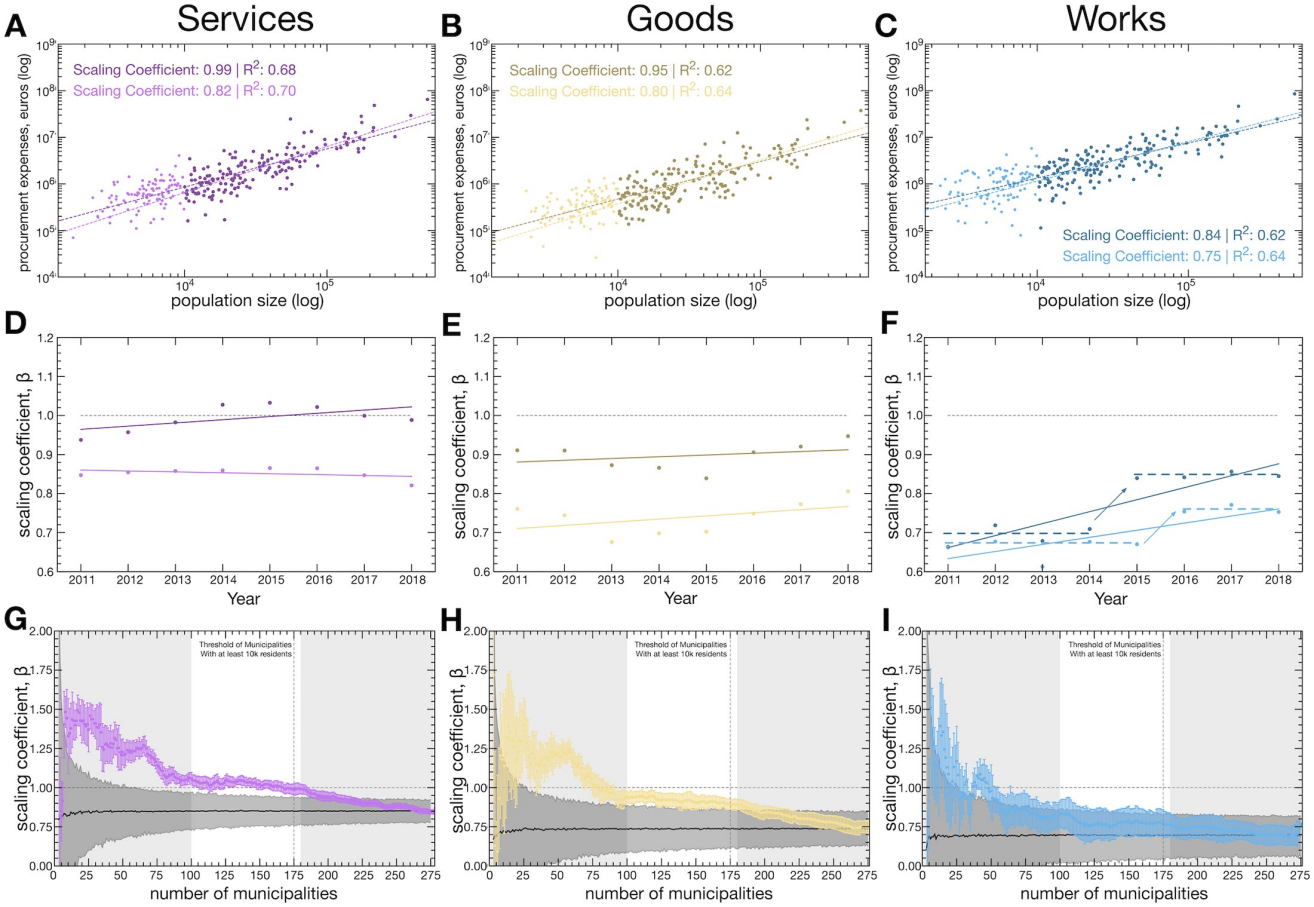
Decomposition of the scaling factors evolution per procurement contract category. (A–C) Relationship between total procurement expenses, by category, in 2018. (D–E) Scaling coefficient per year for each contract category. (A–F) lighter colors indicate the analysis conducted on all Municipalities, darker colors on the subset of municipalities with a population greater or equal to 10^4^. (D–F) lines indicate the best linear model but should only serve as a guideline as they are not statistically significant at p-value threshold of 0.1. (G–I) Robustness checks on the estimated scaling coefficients. Colored points show the estimated coefficient when different population thresholds are applied (we do so by adding the least populated municipalities, from the left to the right side, to the sample). Error bars indicate the standard deviation of the estimated scaling coefficient. The black line indicates the average coefficient from bootstrapping the estimation from X (with X from 3 to 275) municipalities selected randomly with replacement, so municipalities could be considered more than once in each sample, and estimate the scaling coefficient of the sample (dark shaded area show the 95% confidence interval). We repeated this procedure 1, 000 times for each value of X. The vertical dashed line indicates the point at which added municipalities have a population lower than 10^4^.

Fig 3 extends the analysis done in Fig 2B and 2C to different procurement contract categories: Services, Goods, and Works. Like in Fig 2, Light colors refer to the entire set of municipalities, while darker colors represent the sample of municipalities with a population size larger than 10^4^. Fig 3A–3C show the scaling relationships in the year of 2018 for all three categories of procurement contracts. As with the results in Fig 2C, Goods and Works contracts show a sublinear scaling. In contrast, Services show an almost linear relationship but only if the most populated municipalities are considered, and a sublinear relationship when the entire set is analyzed.

Now, focusing on the coefficients obtained for the entire set of municipalities. Public procurement contracts are, in general, associated with the costs of city governance: maintenance, expansion, and functioning of public infrastructures. In other words, they correspond to infrastructure costs. In that sense, the sublinear behavior is inline with past results from urban scaling laws literature. Moreover, the sublinear behavior suggests that we are in presence of an economy of scale. That is, larger municipalities only need to spend a fraction of the *per capita* value spent by less populated municipalities in their functioning.

Fig 3D–3F show that, unlike the results in Fig 2B, the annual upward trend of the scaling coefficient is absent in Goods and Works contracts (note that the slope coefficients estimates in all trend lines are non-significant at a significance level of 10%). However, in the case of Works contracts, these exhibit a transition, around 2014/16, between two seemingly stable regimes, a behavior that contributes to understanding the upward trend identified in Fig 2B. The transition observed in the Works contracts’ scaling coefficient implies that, after 2014/15, the most populated municipalities started to spend proportionally more than less populated ones, albeit the persistence of a sublinearity character still means that less populated municipalities spend more on a *per capita* basis. But what can explain such an abrupt change in the coefficients of the Works contracts?

Between 2013/15 several events took place that can help us better understand the context in which the above-mentioned transition took place. Portugal left the Troika bailout program in 2014 [63]. The program is notorious for having introduced unpopular policies to control public sector finances. Moreover, the period is marked by two nationwide elections—the municipal elections of 2013 and the parliamentary elections in 2014—that lead to a shift in the political landscape from center-right to center-left [64–66]. Arguably, such change could have contributed to a shift in the philosophy of public investments. Economically, 2014 is marked by an increase in private sector activity accompanied by a steady rise in tourism flows to Portugal [67, 68]. Although contextually important, it is difficult to pin these events to the underlying cause of the transition in the scaling coefficients of Works contracts. Instead, they help explain the steady decline in procurement activity during the bailout program and the observed rise afterward (see Fig 1A and 1B), as it became easier for public administration to obtain funding.

However, a more significant event took place in 2014. A new municipal finances law, which entered into practice on January 1st, 2014, tightened the ability of municipalities to contract debt. Interestingly, at the time, it was widely speculated that the new law would put particular pressure on small municipalities’ ability to finance their investments [69–73]. Naturally, such uneven pressure could have led to the behaviour observed in Fig 3F: a decrease in the procurement activity inversely proportional to the population size increases the slope of the relationship. It is also reasonable to understand that such law would manifest particularly in some contract categories rather than others: Works contracts are linked with construction projects and public investment in infrastructure; while Goods and Services contracts are associated with the regular functioning expenses of the municipality (e.g., engineering services, finance and accounting services, training and development, furniture, IT equipment, books, vehicles, medical supplies and other commodities) where cuts are less likely to occur.

We conclude this section by testing the robustness of the scaling coefficients by setting different population thresholds and by bootstrapping the coefficients’ estimation.

Fig 3G–3I explore the impact on the estimated scaling coefficient (Y-axis) by considering only the *n*^*th*^ (X-axis) most populated municipalities (colored points with error bars) or by considering a random sample of *n* (X-axis) municipalities (black curve) (results correspond to the average from 500 independent samples. Each sample is done by first selecting a year at random, and then *n* municipalities also at random). When performing a threshold by population size, we observe three distinct regimes: First, when only the most populated municipalities are considered (left-hand shaded area Fig 3G–3I), there is a high variance in the estimated scaling coefficients; Second, an intermediate regime in which the coefficient remains stable to variations in the number of municipalities (center white area Fig 3G–3I); Third, a regime of linear convergence of the coefficient (right-hand shaded area Fig 3G–3I). In the second and stable regime we qualitatively observe the same relationship between the estimated coefficients of the three categories of contracts: Services with a higher coefficient close to one while Works has the lowest coefficient around 0.75. Also important to note, the threshold of 10^4^ inhabitants is located in the rightmost boundary of stable regime (*x* = 175, vertical dashed line). Moreover, these regimes that appear when filtering the municipalities by population size are absent when the coefficients are estimated by performing random samples of similar size, where we recover the coefficient estimated from the entire set of municipalities (black curve in Fig 3G–3I).

In summary, different population size thresholds can lead to differences in the estimated coefficients of Services, Goods, and Works (see Fig 3D to 3I), yet, our results are robust and unbiased to random samples of different sizes, meaning that the data points are equally distributed in the entire domain of analysis. Moreover, since our goal is to obtain a comprehensive picture of the regional dynamics of Portuguese municipalities, one that is relevant to assess policy implications of procurement activity, we focused the analysis on the entire set of municipalities.

### Municipal procurement Scale-Adjusted Indicators

One major challenge when developing a comparative analysis of regional units relates to how an indicator scales along the dimension of analysis (e.g., region area, population size, etc.). For instance, it is common to compare regions on a *per capita* basis. However, such comparison relies on the implicit assumption that indicators scale linearly with population size, therefore the estimators will be inconsistent under sub or superlinearity regimes, in which case outcomes can suffer from increasing/decreasing returns with the population size. Since that is not the case for most indicators, see Fig 2, we can end up with erroneous conclusions. In that sense, urban scaling laws literature proposes using the residuals of each region from the specific scaling law as a reference model.

We follow by estimating the so-called *Scale-Adjusted Indicators* (SAI) [11, 34, 74] to quantify deviations of each municipality procurement activity from the scaling reference model. The SAI correspond to the residuals, which are computed as

$$\begin{array}{c} {SAI}_{i,t}=log_{10}\frac{Y_{i,t}}{Y(N_{i,t})} \end{array}$$ (2)

where *Y*_*i*,*t*_ is the observed expenditure of municipality *i* on year *t*, and *Y*(*N*_*i*,*t*_) is the predicted value given the population size of such municipality. Unlike *per capita* indicators, the SAI are dimensionless and independent of population size [11, 34, 74]. The SAI capture human and social dynamics specific to a given place and time, allowing for a population-unbiased comparison between regional administrative bodies. For instance, SAI have been used to identify clusters of regions with similar activity patterns [11]. In our case, the SAI provide a quantitative unit of comparison between municipalities in regards to how much their expenditure, in different categories of procurement contracts, deviates from what would be expected from a municipality of a given dimension. Hence, allowing us to account for non-trivial effects due to population agglomeration while being a more accurate of cross regional comparison than the more traditional per-capita-based metrics.

Hence, a municipality exhibiting a positive SAI (> 0) in a particular procurement contract category implies that it spends more than it would be expected for that category given its population size. Likewise, a negative SAI (<0) means that a municipality spends less than it would be expected given its population size. For instance, SAI can be used to compare municipalities along the different categories of contracts and estimate their similarity in terms of procurement activity patterns while controlling for differences in population size.

**Fig 4:**
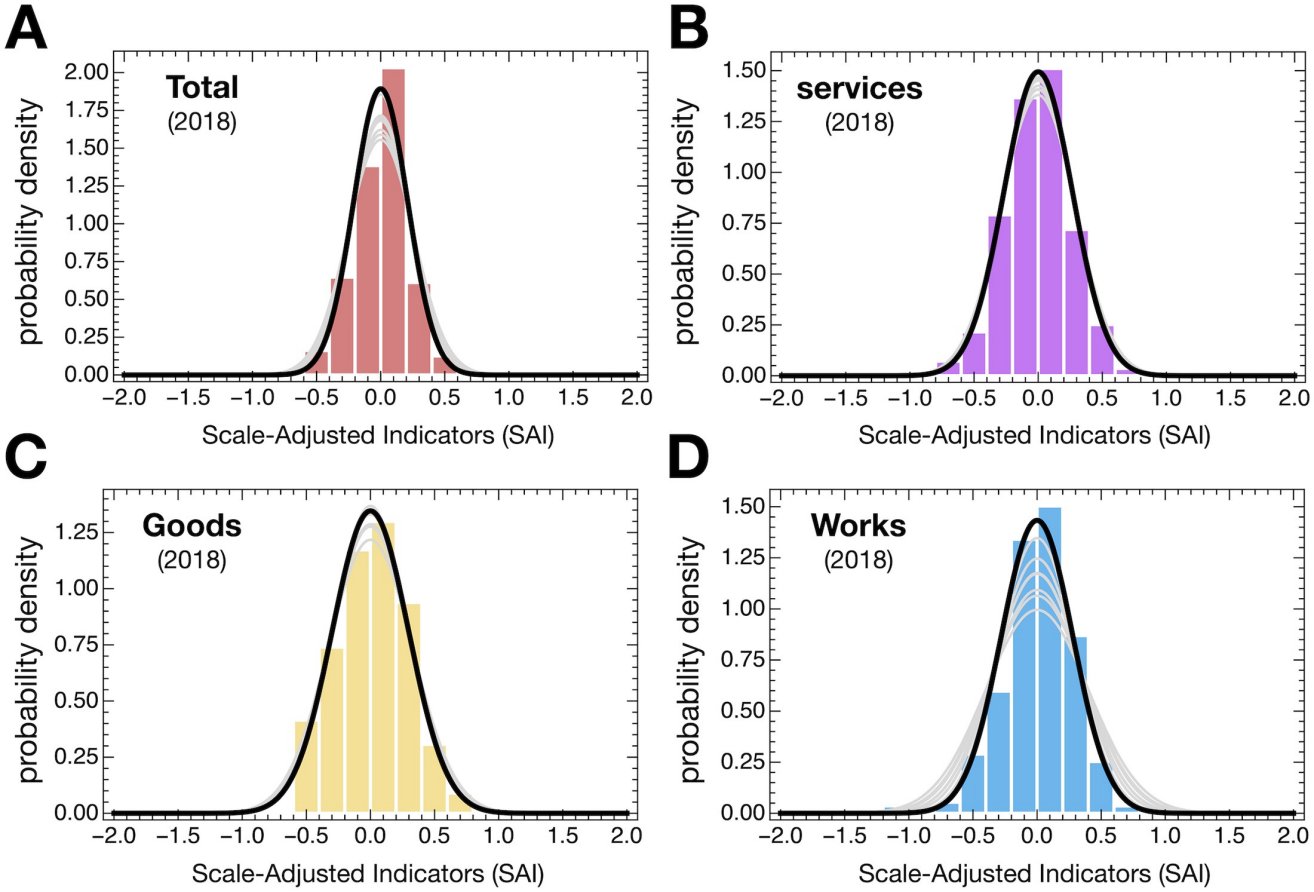
Distributions of Scale-Adjusted Indicators (SAI). Bars show the distribution of SAIs for 2018; curves show the best fitted Normal Distribution to the SAI data for each year. Except for the observations for one year and one category of contract (Goods in 2014) the hypothesis that SAI follow a normal distribution cannot be disproved using the Cramér–von Mises criterion at the significance level of 5%. The map shapefile was sourced from www.dados.gov.pt.

Fig 4 shows the distribution of SAI obtained for different categories of contracts for 2018 and the best fit Normal Distribution for the SAI estimated per year. In all but one case, SAI are Normally Distributed. Moreover, the SAI are uncorrelated with population size and show no heteroscedasticity.

### Regional divide

Over the past century, scholars have looked to characterize existing regional profiles Portugal from either traditional and/or modern sociological considerations [75]. These profiles have shaped much of the regional political debate in Portugal. They are commonly found at the center of policies designed to mitigate the development gap between rural (impoverished and low populated) and urban (more affluent and densely populated) areas. The two most popular divisions/profiles we consider here, divide Portugal in North/South and Coastal/Interior regions [75]. The North and South divide centers around historical and cultural differences between regions that impacted their spatial organization differently [76]; while the Coastal and Interior divide concerns a more modern rationale based on differences in economic development and the ensuing migration patterns observed over the past century [75, 77–82]. In this section, we compare these regional divisions of Portugal and characterize them according to the procurement activities of the municipalities that make up each [11, 83]. In doing so we attempt to answer the question of whether there are distinguishable procurement activity patterns between Portuguese regional divisions.

**Fig 5:**
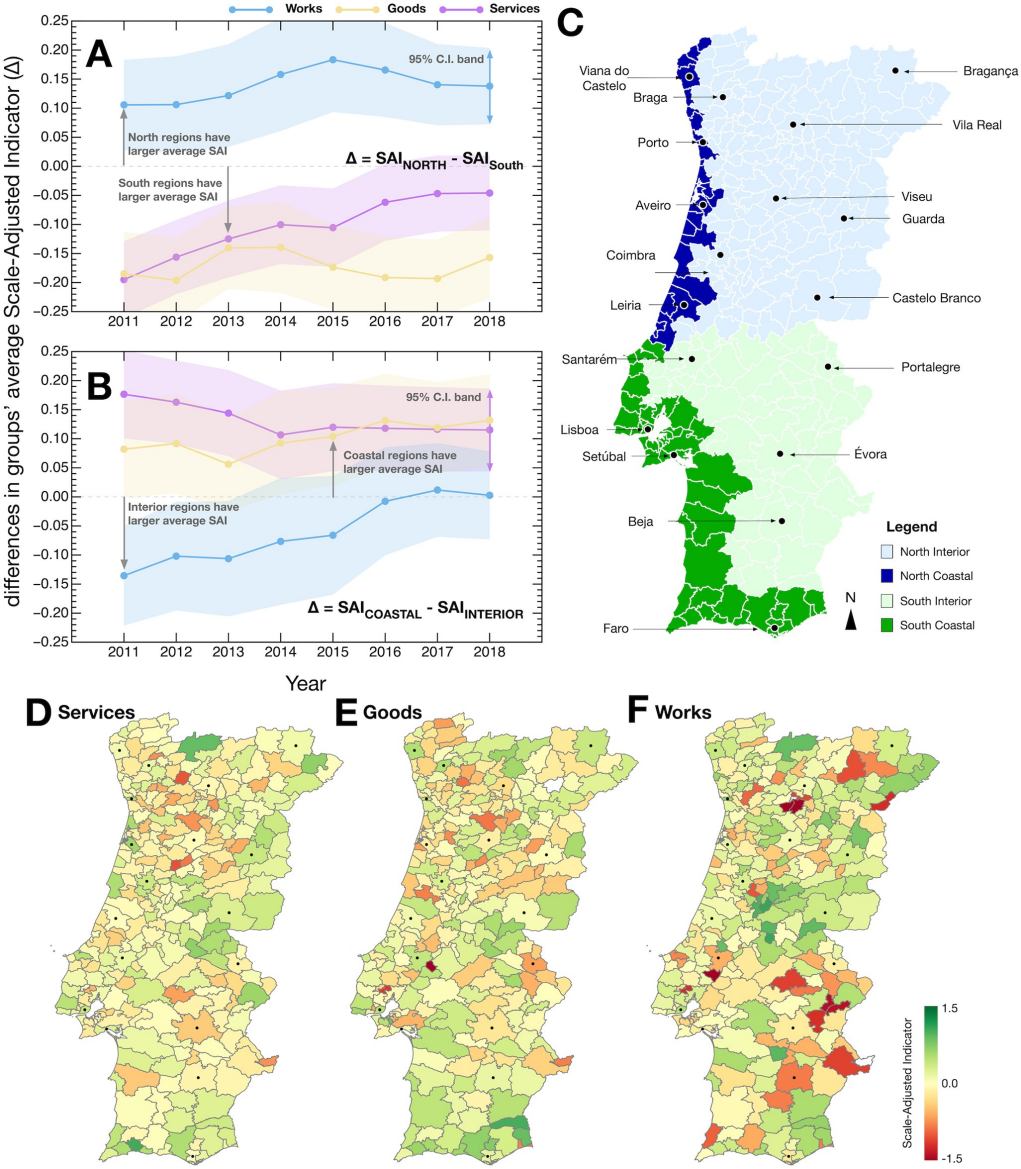
Annual differences in the Scaling-Adjusted Indicators between different groups of Portuguese municipalities. (A) Differences between North and South. (B) Differences between Coastal and Interior. (C) Portuguese municipalities colored according to the groups to which they were assigned. District capitals are indicated. (D–F) show the Scale-Adjusted Indicators of each municipality for each procurement contract category in the year 2018. Minimum and maximum of the color range are set to the maximum absolute Scale-Adjusted Indicator observed. In (A) and (B) the shaded areas correspond to the 95% confidence interval band of the difference between two independent samples with continuous outcomes. The map shapefile was sourced from www.dados.gov.pt.

To that end, and to test the validity of divisions discussed in the literature we grouped municipalities according to whether they are located on the Coast/Interior or in the North/South (it is noteworthy to mention that despite the heated sociological literature on the regional divisions in Portugal, we did not find a generally agreed-upon definition of these regional groups nor a pre-existing annotated dataset at the municipal level). We defined as Coastal municipalities all those that have a coastline or enclaves of municipalities with a coastline, else they were categorized as Interior. Moreover, we used the coordinates of each municipality city center as a point of reference to classify them as being in the North or South. In particular, we classified as North the 140 municipalities whose city coordinates are the northernmost; the remaining 138 were classified as being in the South. Fig 5C shows the classification of each municipality.

Fig 5A compares the average SAI between North and South municipalities (Δ = SAI_NORTH_ − SAI_SOUTH_) and Fig 5B repeats the analysis between Coastal and Interior municipalities (Δ = SAI_COASTAL_ − SAI_INTERIOR_). The analysis of the differences in SAI are indicative of systematic differences in the execution of public funds among groups, as such they allow us to identify whether, for instance, a specific group tends to spend on average more in a particular category of procurement contracts than the other. In other words, we are comparing differences in the observed deviations. The differences in SAI between the regional groups—Northern/Southern [84] or Coastal/Interior [81]—reveal that there are distinguishable differences between regions. For instance, Northern municipalities tend to exhibit, in average, larger SAI in Works, while Southern Municipalities are characterized by larger SAI in Services and Goods. These patterns remain qualitatively the same over the years. Moreover, Coastal municipalities tend to have larger SAI in Goods and Services procurement contracts, while Works contracts have evolved from being larger in the Interior to reach parity since 2016. In the SI we show the average procurement activity patterns for each of these regions.

### Profiles of procurement activity

Despite the identified differences between North/South and Coastal/Interior, the spatial distribution of SAI does not directly map into such regional groups (see Fig 5D–5F). Instead, they exhibit a richer spatial distribution of procurement patterns, which often breaks geographical proximity.

Using the SAI, we can identify alternative regional divisions grounded in the comparison between procurement activity. In that sense, municipalities can be grouped according to their matched procurement activity profiles. Here, and in order to exemplify it, we define a profile as the combination of the SAI signs from each of the procurement contract categories: Goods; Services; and Works. Then, each profile identifies whether a municipality presents positive/negative deviations in expenditures of one, two, three, or none of the procurement categories.

**Fig 6:**
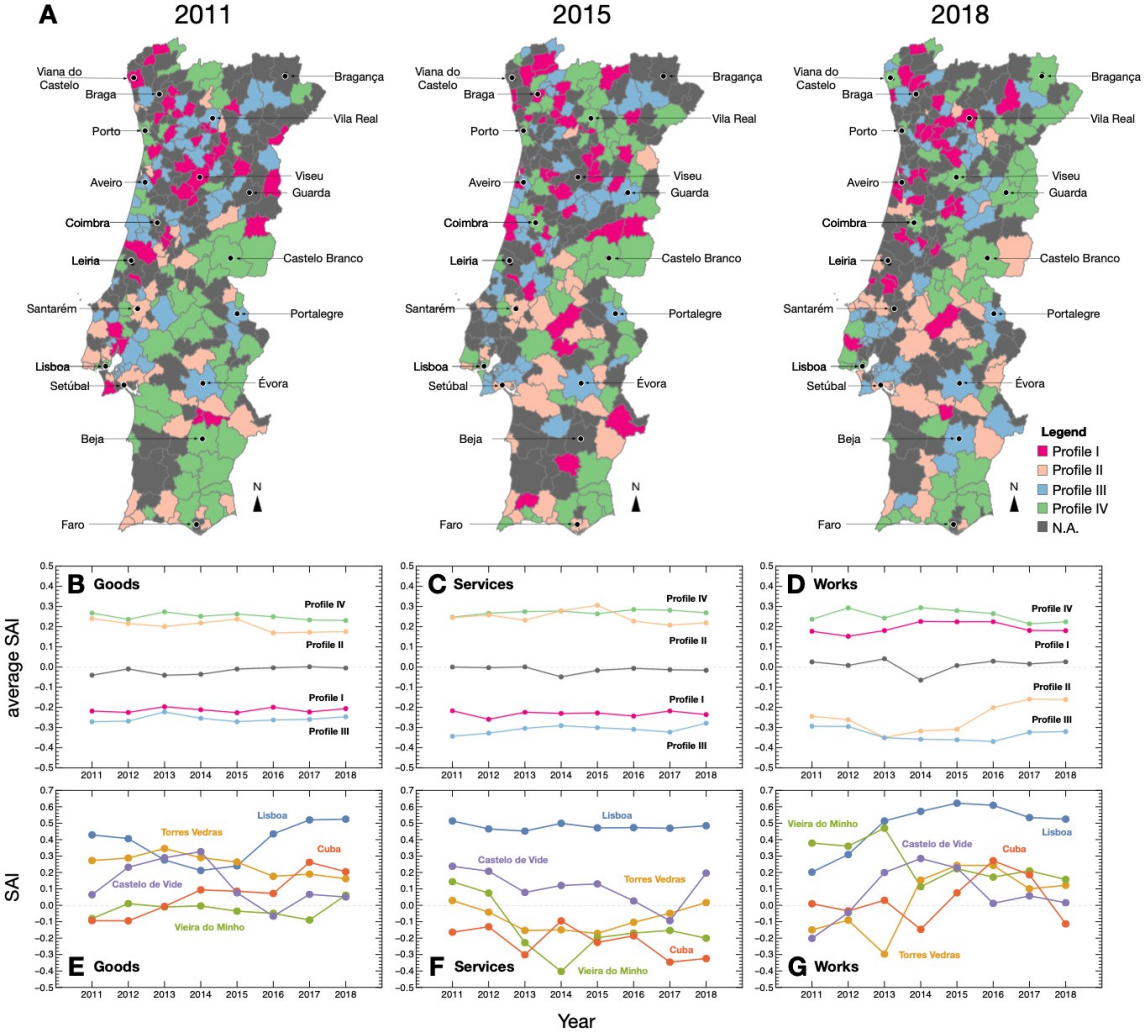
Procurement activity profiles. (A) Spatial distribution of profiles in 2011, 2015, and 2018. Profile I (red) concerns municipalities that exhibited positive SAI in works with negative SAI in goods and services; profile II (light orange), includes municipalities with positive SAI in goods and services and negative SAI in works; profile III (blue) and profile IV (green) represent municipalities with all SAI, respectively, negative and positive in the three procurement categories. Municipalities not assigned (N.A.) to a profile are colored in dark gray. (B–D) Average scale adjusted indicators (SAI) in each procurement category for the municipalities associated with each one of the four highlighted procurement activity profiles. (E–G) Example of temporal evolution of the SAI for each procurement category for five municipalities representative of the different population scales: Lisboa (10^5^.7); Torres Vedras (10^4^.89); Vieira do Minho (10^4^.08); cuba (10^3^.67); Castelo de Vide (10^3^.47).

Table 1 summarizes the possible profiles (in terms of the SAI signs in each category) and their prevalence in a typical year. It shows that four profiles are particularly dominant (in bold) exhibiting a prevalence larger than we would expect from a random association between municipalities and profiles. These four profiles also account for approximately 66% of the observations. The four dominant profiles can be summarized as follows: profile I includes municipalities that exhibited positive SAI in works and with negative SAI in goods and services; profile II includes municipalities with positive SAI in goods and services and negative SAI in works, profile III considers municipalities with negative SAI in all three categories; and finally profile IV considers municipalities with positive SAI in all categories. Fig 6B–6D, show the average SAI of the municipalities for each contract category in each of the four profiles plus the remaining municipalities not assigned to a profile (referred to as N.A.). These figures allow to compare profiles not only according to their signs but also their respective magnitudes.

**Table 1:** Summary of the prevalence of each of the eight possible procurement activity profiles. In bold, the profiles with a high prevalence and which are discussed in more detail in the main text. Each profile is characterized by the sign of the SAI in each of the procurement categories: Services; Goods; and Works. The prevalence corresponds to the fraction of observations (i.e., fraction of municipalities) in such profile. The four identified profiles—I, II, III, and IV—account for approximately 66% of the observations.

ID	Profiles			Prevalence
	Services	Goods	Works	
**IV**	-	+	+	**0.223**
–	-	+	+	0.103
–	+	-	+	0.099
**II**	+	+	-	**0.125**
**I**	-	-	+	**0.140**
–	-	+	-	0.062
–	+	-	-	0.074
**III**	-	-	-	**0.172**

Fig 6A shows three examples of the spatial distribution of the four profiles under consideration in 2011 (left), 2015 (middle), and 2018 (right). It shows that while in some cases municipalities exhibit the same profile during the entire time window, there are cases in which municipalities undergo changes in their profile. Transitions between profiles are seemingly not random; instead they seem to follow a specific structure, see S4 Table in S1 File, with some transitions being more frequent than others. In particular, we found that transitions between profiles in which only one SAI of the sign changes are more likely to occur. These suggests, that the evolution in municipality procurement activity does not suffer dramatic changes, but instead follows from changes in different categories.

Fig 6E–6G shows the temporal variations in the SAI for 5 municipalities that are representative of the range of population sizes in our sample (see S6 Fig in S1 File for all municipalities). While in the vast majority of the cases variations are stable and remain constant, there are instances in which variations occur around zero and cases in which there more abrupt variations, which are particularly evident in the Works category. Such variations in the Works can be partly explained by the nature of such contracts, which often involve the execution of large construction works that are less frequent and might have a periodicity larger than the level of temporal aggregation analysed. Overall, given the small time-window of analysis we will not deepen the study of municipality-level temporal variations of the SAI, as the current dataset would not allow for generalizable conclusions. Instead, we discuss how the emergent distribution of profiles match with the previous regional divisions and their rationale. Hence, the question being, can we identify common characteristics between municipalities that share similar procurement activity patterns?

The groups of municipalities according to their procurement activity profile, largely break the spatial homogeneity of the regional divisions studied above (North/South and Coastal/Interior). Supporting the idea that such divisions do not translate well into the diversity of profiles from a procurement activity perspective. Profiling the groups enables to reveal common patterns and similarities in governance within groups but also the differences between groups.

Municipalities associated with profiles I and II are characterized by geographical, political, and industrial differences and project the archetypes discussed above along the North/South divide. For instance, profile I is mainly concentrated in the North (dominated by an electoral preference for center-right parties, see SI) and profile II in the center/south (dominated by left and center-left political parties, see SI) [66]. These regions are also known to have different industry structures [84]: The North being more manufacturing intensive, while the south is more Agriculture intensive.

The two remaining groups unveil differences stemming from policy and financially related constrains. Profile III comprises municipalities with a higher debt per capita than the average (see SI), which helps explain the lower public procurement activity of this profile. In contrast, profile IV comprises a set of municipalities that have been able to capture more funds from the European Union on a per capita basis (see SI) while having relatively low debt. Thus, their ability to execute more procurement than expected in all categories of contracts is not surprising. These findings may indicate the presence of self-reinforcing mechanisms (profiles I and II), that is, by means of rooted cultural and context-specific patterns of public procurement activity. However, a more robust analysis of the factors underlying such differences is needed to adequately identify the socio-cultural and political factors driving the prevalence of the different procurement activity profiles. Moreover, clustering techniques aimed at grouping municipalities along the revealed SAI patterns and their temporal dynamics could provide a more complete characterization of existing regional differences. Nevertheless, it is clear that SAI provides a significant dimension for the accurate identifications of regional (dis)similarities, one that controls for differences in population size.

## Conclusions

Having the correct methodology to perform a comparative regional analysis of procurement activities is critical to evaluate the effectiveness of public policies and their socio-economic consequences. However, it is a challenge to develop accurate measures when indicators do not scale linearly with, for example, population size. Here, we proposed using methods borrowed from urban scaling laws to analyze procurement activities among 278 Portuguese municipalities by different contract categories.

We characterised the scaling coefficient of procurement activity and put it at a glance with an array of other indicators (Fig 2). Municipal procurement activity tends to scale sublinearly with population size, meaning that increasing the population size lowers the value spent *per capita* in public contracts. Such behaviour is true for both the total value spent in public procurement and among the different categories of contracts (Figs 2 and 3).

We observe an upward trend in the annual variation of the scaling coefficients. However, we argue that such a trend is being modulated by the observed transition in the Works procurement contracts that jumped from ≈0.7 to ≈0.85 between 2014/16. We link such transition to several socio-political events that took place in Portugal but, more importantly, by a revision of the municipal finances law that entered in practice in January 1st, 2014.

Finally, looking at the deviations from the scaling laws (the SAI), we compared differences in the revealed procurement activity between regional groups of interest—North/South and Coastal/Interior—and demonstrate the potential of using the SAI in the definition of new groups based on their similarity in procurement activity. The showcased example focuses on four groups of municipalities that present differences in procurement activity profiles. These groups are also associated with differences in electorate political preferences and industrial structures (profiles I and II), but also by different financial constrains (profile III reveals indebted municipalities) or policy interventions (profile IV are revealed to have received higher EU funds). We believe that these findings justify the potential of the current framework for study procurement activity and open the doors for future research aimed at understanding and comparing regional dynamics within and between countries.

Although previous works have analyzed procurement data [8], they have done so using relatively smaller samples, which limited the investigation of universal governing patterns at any level. Ongoing work looks at developing of a more robust model to understand the link between public procurement activity at the regional level, and economic development. A challenge that requires identifying the appropriated indicators and the adequate model specification [85]. Moreover, future research may also extend this analysis to more fine-grained of contract category classifications (i.e., exploring the CPV classification) but also to other countries and regions, particularly European public procurement repositories. The latter would allow us to validate the identified scaling relationships and reveal differences in behaviours across countries with diverse administrative processes and cultural contexts.

## Supporting information

S1 File Contains all the supporting tables and figures. (DOCX)
