# Supplementary material for: Scaling behavior of public procurement activity
Source: PLoS One. 2021 Dec 8;16(12):e0260806. doi: 10.1371/journal.pone.0260806 (PMC8654174; doi:10.1371/journal.pone.0260806)
Supplement: S1 File — (DOCX) [file pone.0260806.s001.docx]

**Supporting Information**

**Scaling Behavior of Public**

**Procurement Activity**

António Curado^1^, Bruno Damásio^1,2^, Sara Encarnação^3,4^,
Cristian Candia^5,6,7,8^, and Flavio Pinheiro^1^

^1^ NOVA Information Management School (NOVA IMS), Universidade Nova de Lisboa, Campus de Campolide, 1070-312 Lisboa, Portugal

^2^ Instituto Superior de Economia e Gestão (ISEG) - Universidade de Lisboa, 1700-284 Lisboa, Portugal

^3^ Interdisciplinary Centre of Social Sciences (CICS.NOVA), Faculty of Social Sciences and Humanities (FCSH/NOVA), 1069-061 Lisboa, Portugal

^3^ ATP-group, P-2744-016 Porto Salvo, Portugal

^5^ Data Science Institute, Facultad de Ingeniería, Universidad del Desarrollo, Las Condes, 7610658, Chile.

^6^ Kellogg School of Management, Northwestern University, Evanston, IL 60208, USA

^7^ Northwestern Institute on Complex Systems (NICO), Northwestern University, Evanston, IL 60208, USA

^8^ Centro de Investigación en Complejidad Social (CICS), Facultad de Gobierno, Universidad del Desarrollo, Las Condes 7550000, Chile

# Overview

This document provides supplementary information that extends and support the analysis conducted in the main manuscript. The document is organized as follows, each section focusses the discussion on a particular topic of analysis, the text is in the initial pages while supporting Figures and Tables can be found in the final pages of this document.

**Public Procurement dataset**

The Portuguese public procurement open data repository is managed by **Instituto dos Mercados Públicos, do Imobiliário e da Construção, I.P. (IMPIC**) and can be consulted through the portal [www.base.gov.pt](http://www.base.gov.pt). Data follows the Open Contracting Data Standard (<https://www.open-contracting.org/data-standard/>), which is an unified procurement format that makes it easier for researchers to analyze data across different countries datasets.

The dataset comes in a tabular format, with each row concerning one contract and the available features in the columns. We have focused in give specific features:

- 'buyer.id' - Fiscal identifier of the procuring entity;
- 'tender.contractPeriod.startDate' - Start date of the contract;
- 'tender.value.amount' - The value of the contract;
- 'tender.mainProcurementCategory' - The main category, see **Table S1**;
- 'tender.items' - Column containing one or multiple common procurements codes(CPV) for the given contract. See **Table S2**.

**Figure S1** shows the total volume in Euros and number of contracts per procurement type.

# Additional data

As mentioned in the main manuscript, the procurement dataset was enriched with additional data sourced from two main sources: *Instituto Nacional de Estatística* (INE) and PORDATA. **Table S3** summarizes some descriptive statistics of each one of these datasets, in particular the time interval of available data, if it contains missing values, the unit of the variable and corresponding descriptive statistics (the mean, the standard deviation, the minimum, and the maximum).

Data obtained from PORDATA portal is publicly available. However, data sourced from INE data it was obtained through a research protocol, thus is not public and cannot be shared due to privacy reasons. Researchers interested in analyzing such data should reach out to INE through e-mail [info@ine.pt](mailto:info@ine.pt) and inquire about the conditions to access the data.

# Link between Procurement Activity and Economy Size

One relevant question when studying municipal procurement contracts data concerns its link with other economic dimensions. In other words, does the procurement activity introduce new valuable information or does it function as a proxy for the size of the economy?

**Figure S2** shows the relationship between current expenses of municipalities and total procurement expenses. Each data point represents a municipality in a year. In aggregate we can observe a high linear relationship between the two quantities, with a Pearson correlation of 0.97. Moreover, year-by-year correlation averages 0.88, with a minimum of 0.80 (2011) and a maximum of 0.95 (2016).

In that sense, evidence suggests that procurement expenses can constitute a proxy to assess economic activity of municipalities. As we argue in the main text, the analysis of procurement contract data additionally offers several elements that provides it more detail on the nature of the expenditures, besides information about all parties involved, which, in our opinion, justifies its study and understanding.

# Regional Analysis

**Figure 5** of the main text shows the differences between the procurement activity of two different groups of regions: North versus South; and Interior vs Coastal. Here, we show the underlying procurement activity patterns of each of such regions, see **Figure S3**.

# Procurement Activity Profiles

One of our findings is that municipalities change their profiles dynamically over time. However, they do not do it at random. Instead, they follow a well-structured sequence of transitions between profiles. We measure it by computing the fraction of times it was observed that a municipality transitioned from one profile to another between two consecutive years. **Table S4** summarizes the findings, highlighted are the transitions with values above what we would expect to observe in an unfirm process (i.e., one in which transitions are purely at random). For all purposes, the matrix in **Table S4** represents the transitions of a Markov process, in which each state represents each of the eight different profiles. Last column of **Table S4** shows the stationary distribution of the ensuing process, which provides an indication of what would be the expected prevalence of each profile if the inferred matrix of transitions translates a mechanistic process. One interesting finding is transitions are more likely to occur between profiles in which only one of the SAI changes signs. In other words, changes in procurement expenditure are not abrupt, but instead evolve smoothly. Moreover, from the stationary distribution five profiles have a high expected prevalence, and four match the dominant profiles discussed in the main text.

It is important to note that although transitions between states are not to be expected with high frequency, they do naturally appear as municipalities adjust the way they execute their procurement activities. In some cases, transitions occur as noise from time-series that vary around zero. In other cases, they are just the manifestation on the changes in procurement behavior in municipalities. Regardless, the emergent behavior of all municipalities is robust over the time-window of analysis as shown in Figure 2 of the main manuscript.

# Procurement Activity and Regional Economic Activity

In this section we briefly inspect how procurement activity relates to regional economic activity, which we capture through a wide range of indicators commonly used to estimate purchasing power^[[1]](#footnote-1)^. To that end, we estimate the Pearson correlation between the SAI associated with procurement activity by contract type -- Services, Goods, and Works – and the SAI obtained from regional economic indicators such as the average salary of full-time works; self-reported individual gross income; total volume of ATM withdrawals; total value collected from Municipal property tax; the number of workers; total amount of loans; Volume of Business activities in Accommodation, Catering, and Retail (excluding car sales).

**Figure S5** shows the annual correlations between the procurement SAI and regional indicators. In all panels, the Y-axis indicates the Pearson correlation, and X-axis represents the year of analysis. Each curve's color indicates the type of procurement contract, and each panel depicts the results for a particular regional economic activity indicator. Surprisingly, Works procurement contracts exhibit null to negative correlation in most indicators. Exceptions are in the number of workers and the total volume of loans. This interesting finding raises questions on the impact of public procurement work contracts as an effective policy instrument that we believe deserves further research. These results show that different types of public procurement might spill over to different economic dimensions in different ways. In contrast, Services and Goods procurement contracts show a stable positive correlation with most indicators.

More importantly, these results suggest that there exists a link between procurement activity and the regional economy. In that sense, such economic indicators can be used effectively as monitoring proxies for the evaluation of public policy programs. Moreover, the short analysis also supports the potential of using procurement SAI as an explanatory variable in models that look to understand the importance and relevance of different types of public procurement activity in the local economy.

Finally, these results and ensuing discussion are preliminary and serve to show the potential application of SAI in enriching models that aim at characterize the development and evolution of Portuguese municipalities.

# Temporal Evolution of SAI

In this section we briefly discuss the stability of the temporal variations of the SAI in the procurement contracts of the three main categories discussed in the main text: Works, Services, and Goods.

Figure 6E, 6F, and 6G of the main manuscript show the temporal evolution of five municipalities representative of the population size range. It shows how Works procurement contract categories exhibit larger year-to-year variation than when compared with the Goods and Services categories. The larger variations in Works procurement contracts can result from the nature of these contracts. For instance, they often correspond to large construction works that are rare and will have a large impact in a particular year. Moreover, it can also be the case that the choice of aggregating Works contracts per year is too temporally fine-grained and below the characteristic timescale of such category. However, the later hypothesis would require a longer time-series to be investigated.

Moreover, we also see that municipalities can undergo transitions between positive/negative SAI to negative/positive values over time. Typically, these changes are smooth, but can also be somewhat more abrupt. However, these conclusions are myopic as we are looking at a small time-window and as such variations can look inflated. Once again, our conclusions are limited due to the limitation in the dataset.

Here, we expand the above observations to all municipalities. Following previous works we show in Figure S6 shows the temporal variations of the SAI for all municipalities. These are shown to be stable (i.e., we do not observe with high frequency breaks in the temporal evolutions) and to be within a limited range of values.

Moreover, Figure S7 shows the cumulative distribution of year-to-year variations, showing that the majority of variations are rather small, and that each type of contract category seems to have its own characteristic year-to-year change, with Services changing less and Works changer more.

**Table S1** – Description of the three main types of Public Procurement Contracts according to the UE public procurement guidelines.

| **Work Contracts** | Public contracts having as their objective either the execution, or both the design and execution, of works, for example building or civil engineering works such as a road or sewage plant. |
| --- | --- |
| **Goods Contracts** | Public contracts having as their object the purchase, lease, rental or hire purchase with or without option to buy, of products such as stationery, vehicles or computers. |
| **Service Contracts** | Public contracts other than public works or supply contracts having as their object the provision of services such as consultancy, training or cleaning services. |

**Table S2** – Description of the two-digit first level Common Procurement Vocabulary (CPV) classification of Public Procurement Contracts.

| **ID** | **Description** |
| --- | --- |
| **03** | Agricultural, farming, fishing, forestry and related products |
| **09** | Petroleum products, fuel, electricity and other sources of energy |
| **14** | Mining, basic metals and related products |
| **15** | Food, beverages, tobacco and related products |
| **16** | Agricultural machinery |
| **18** | Clothing, footwear, luggage articles and accessories |
| **19** | Leather and textile fabrics, plastic and rubber materials |
| **22** | Printed matter and related products |
| **24** | Chemical products |
| **30** | Office and computing machinery, equipment and supplies except furniture and software packages |
| **31** | Electrical machinery, apparatus, equipment and consumables; lighting |
| **32** | Radio, television, communication, telecommunication and related equipment |
| **33** | Medical equipments, pharmaceuticals and personal care products |
| **34** | Transport equipment and auxiliary products to transportation |
| **35** | Security, fire-fighting, police and defence equipment |
| **37** | Musical instruments, sport goods, games, toys, handicraft, art materials and accessories |
| **38** | Laboratory, optical and precision equipments (excl. glasses) |
| **39** | Furniture (incl. office furniture), furnishings, domestic appliances (excl. lighting) and cleaning products |
| **41** | Collected and purified water |
| **42** | Industrial machinery |
| **43** | Machinery for mining, quarrying, construction equipment |
| **44** | Construction structures and materials; auxiliary products to construction (except electric apparatus) |
| **45** | Construction work |
| **48** | Software package and information systems |
| **50** | Repair and maintenance services |
| **51** | Installation services (except software) |
| **55** | Hotel, restaurant and retail trade services |
| **60** | Transport services (excl. Waste transport) |
| **63** | Supporting and auxiliary transport services; travel agencies services |
| **64** | Postal and telecommunications services |
| **65** | Public utilities |
| **66** | Financial and insurance services |
| **70** | Real estate services |
| **71** | Architectural, construction, engineering and inspection services |
| **72** | IT services: consulting, software development, Internet and support |
| **73** | Research and development services and related consultancy services |
| **75** | Administration, defence and social security services |
| **76** | Services related to the oil and gas industry |
| **77** | Agricultural, forestry, horticultural, aquacultural and apicultural services |
| **79** | Business services: law, marketing, consulting, recruitment, printing and security |
| **80** | Education and training services |
| **85** | Health and social work services |
| **90** | Sewage, refuse, cleaning and environmental services |
| **92** | Recreational, cultural and sporting services |
| **98** | Other community, social and personal services |


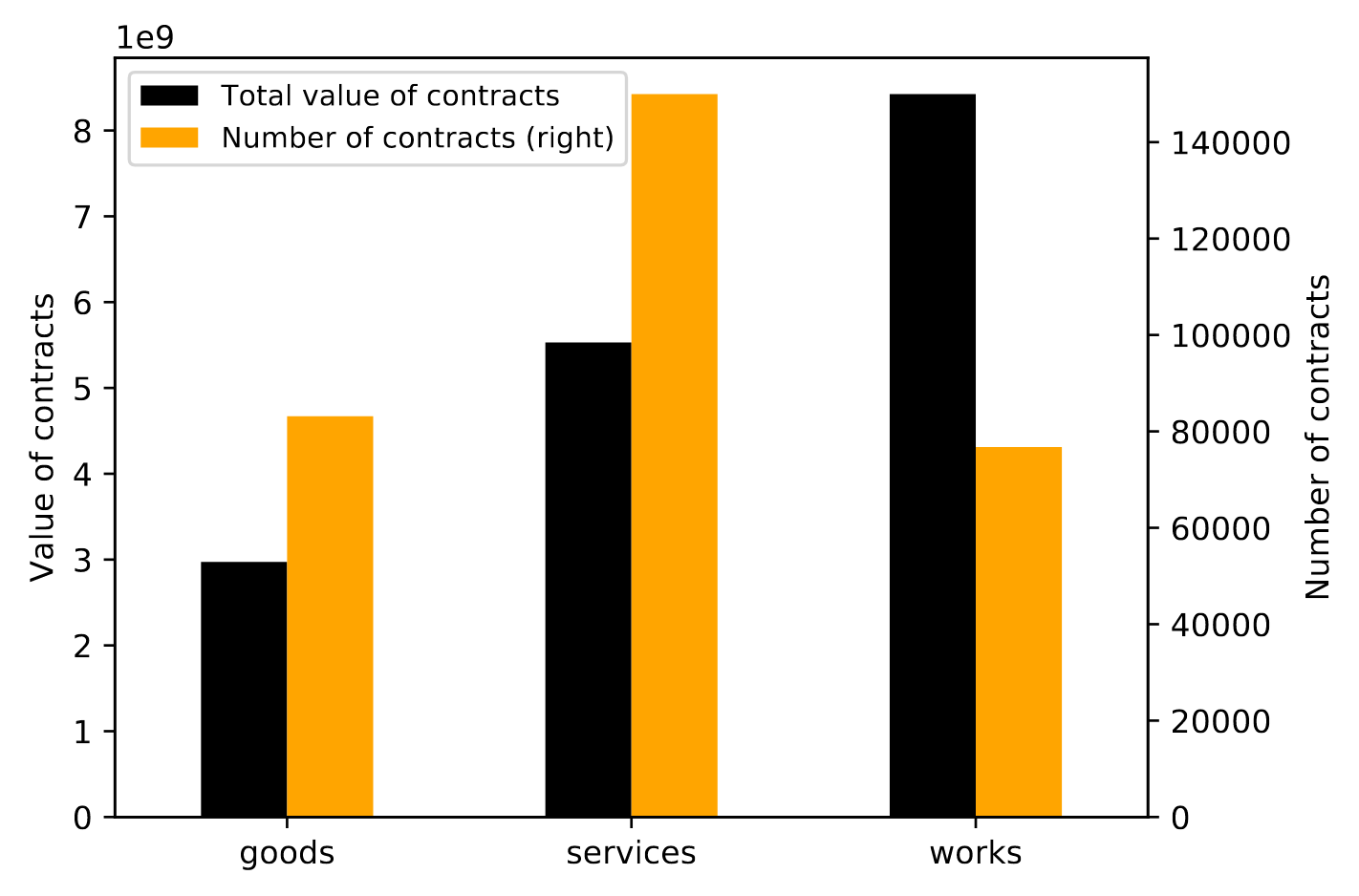


**Figure S1** – Number of contracts and total value aggregated by main contract category

**Table S3** – Descriptive Summary of the Additional Variables used to enrich the dataset. Data was sourced from two main sources INE and PORDATA. Column M. Val. Indicates whether the dataset contains Missing Values and the Open indicates if the dataset is publicly available or not.

| **Dataset** | **Years** | **M. Val.** | **Open** | **Unit** | **Mean** | **Std** | **Min** | **Max** |
| --- | --- | --- | --- | --- | --- | --- | --- | --- |
| Imports | 2009/18 | 0 | Y | Euro | 2.03E+08 | 1.09E+09 | 0 | 1.83E+10 |
| Exports | 2009/18 | 0 | Y | Euro | 1.59E+08 | 4.66E+08 | 0 | 7.44E+09 |
| Social Integration Income | 2009/18 | 0 | Y | Euro | 1221 | 2755 | 0 | 31315 |
| Energy Consumption | 2009/17 | 0 | Y | kWh | 22101 | 34272 | 1226 | 383252 |
| Total births | 2009/18 | 0 | Y | Nº | 360 | 700 | 0 | 18276 |
| Doctors | 2009/18 | 0 | Y | Nº | 158 | 650 | 0 | 9573 |
| Large Corporations | 2009/17 | 0 | Y | Nº | 4 | 18 | 0 | 285 |
| Culture Atendees | 2009/18 | 0 | Y | Nº | 33086 | 183685 | 0 | 3006910 |
| Divorces | 2009/18 | 0 | Y | Nº | 67 | 135 | 0 | 2143 |
| Amount of Credit | 2009/17 | 0 | Y | Euro | 7.44E+08 | 8E+06 | 0 | 8E+06 |
| House Prices | 2009/18 | 0 | Y | Euro | 52647 | 48297 | 1208 | 575900 |
| Public workers | 2009/17 | 0 | Y | Nº | 428 | 657 | 49 | 10106 |
| Environment Expenses | 2009/18 | 0 | Y | Euro | 2038 | 5448 | 0 | 86858 |
| Total crime | 2009/17 | 0 | Y | Nº | 1259 | 2963 | 15 | 4250 |
| Self-Reported Gross Income | 2011/17 | 0 | N | Euro | 289071 | 672063 | 8574 | 9416926 |
| ATM withdrawals | 2011/17 | 0 | N | Euro | 8.8E+07 | 2E+08 | 3E+06 | 3E+09 |
| Credit given for housing | 2011/17 | 0 | N | Euro | 327468 | 1E+06 | 7356 | 2E+07 |
| Municipal Property Tax | 2011/17 | 0 | N | Euro | 4800 | 10072 | 40 | 118153 |
| Average Income of a full-time worker | 2011/17 | 0 | N | Euro | 895 | 163 | 673 | 2331 |
| Municipality employees | 2011/17 | 0 | N | Nº | 7167 | 19149 | 178 | 295474 |
| Volume of Business Accomodation) | 2011/17 | 90 | N | Euro | 2.6E+07 | 8.3E+07 | 4E+05 | 2E+09 |
| Volume of Business (Catering) | 2011/17 | 0 | N | Euro | 1.6E+08 | 3.4E+08 | 2E+06 | 5E+09 |
| Volume of Business (Retail) | 2011/17 | 361 | N | Euro | 1.6E+08 | 3.4E+08 | 2E+06 | 5E+09 |

**

**Figure S2.** Comparison between procurement expenses and municipal budgets. Panel a) shows the fraction of municipalities with given ratio of procurement expenses and budget. Panel b) linear relationship between procurement expenses and budget.

**Figure S3.** Procurement activity patterns of municipalities located in South and North regions (Left) and in the Interior and Coastal regions (Right).

**Table S4** – Summary of the identified year-to-year transitions between procurement activity profiles. Values in the matrix measure the fraction of transitions from the row profile to the column profile. Goods, Services, and Works columns define the profiles per id. The column Profile indicates the name used to reference such profile in the main text. In bold are the transitions with a value above the value expected from an uniform distribution (1/8). The transition values represent the probability of transition of a Markov Process, in that sense the Sta. Dist. column represent the stationary distribution of the ensuing process, which values rescaled by values expected from a random process.

| **Goods** | **Services** | **Works** | **id** | **1** | **2** | **3** | **4** | **5** | **6** | **7** | **8** | **Profile** | **Sta. Dist.** |
| --- | --- | --- | --- | --- | --- | --- | --- | --- | --- | --- | --- | --- | --- |
| + | + | + | **1** | **0,69** | 0,1 | 0,05 | 0,12 | 0,01 | 0,01 | 0,01 | 0 | IV | **1.019** |
| - | + | + | **2** | **0,21** | **0,5** | 0,02 | 0,02 | 0,08 | 0,1 | 0,01 | 0,05 | N.A. | 0.971 |
| + | - | + | **3** | 0,11 | 0,04 | **0,58** | 0,03 | **0,13** | 0,000 | 0,12 | 0,01 | N.A. | **1.003** |
| + | + | - | **4** | **0,24** | 0,02 | 0,02 | **0,58** | 0 | 0,05 | 0,09 | 0,01 | II | **1.035** |
| - | - | + | **5** | 0,02 | 0,07 | 0,06 | 0 | **0,64** | 0,02 | 0,03 | **0,15** | I | **1.014** |
| - | + | - | **6** | 0,04 | **0,16** | 0,000 | **0,13** | 0,03 | **0,49** | 0,01 | **0,14** | N.A. | 0.994 |
| - | - | + | **7** | 0,02 | 0,01 | 0,17 | 0,11 | 0,04 | 0,000 | **0,44** | **0,16** | N.A. | 0.927 |
| - | - | - | **8** | 0 | 0,02 | 0,02 | 0,02 | **0,14** | 0,05 | 0,06 | **0,7** | III | **1.038** |

**Figure S4.** Characterization of the four groups of municipalities defined by the profiles of procurement. Panel a) compares the fraction of elected representatives per muncipality affiliated with Left and Right wing parties. Independent elected representatives are classified as others. Results are normalized in relation tot he average of all municipalities in ordert o correct for the overall balance of the election. Panels b) and c) compare, respectively) the average per capita Debt and EU funds of municipalities oft he four clusters.


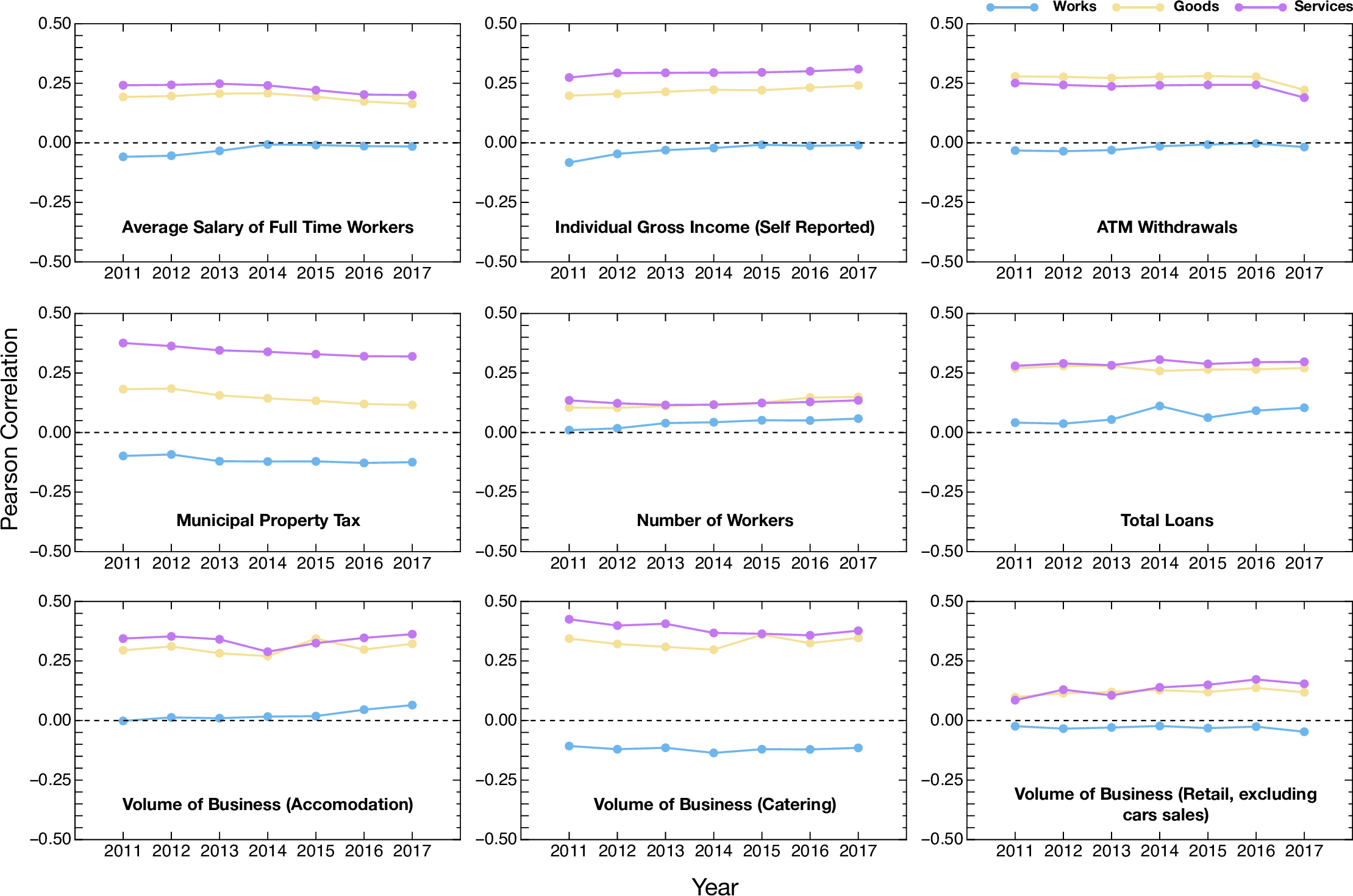


**Figure S5.** Correlations between the Scale-Adjusted Indicators of Public Procurement Activity and Regional Indicators of Economic Activity.

**Figure S6.** Summary of the temporal variations of the SAI for Services, Goods, and Works procurement contracts

**Figure S7.** Cumulative Distribution of the year-to-year variation in the SAI. For reference we add the red horizontal line marks the line of 80% of the observations.

1. Data sourced from Instituto Nacional de Estatística [↑](#footnote-ref-1)
